# Combined Pre- and Post-Emergence Herbicides for Effective Weed Control in Alfalfa (*Medicago sativa* L.)

**DOI:** 10.3390/plants15162461

**Published:** 2026-08-13

**Authors:** Xin-Ran Bao, Yang Gao, Jin-Won Kim, Min-Jung Yook, Do-Soon Kim, Chuan-Jie Zhang

**Affiliations:** 1 College of Animal Science and Technology, Yangzhou University, Yangzhou 225009, China; 19906151359@163.com (X.-R.B.); xx_gaoyang@163.com (Y.G.); 2 National Institute of Agricultural Sciences, Rural Development Administration, Wanju 55365, Republic of Korea; 3 Department of Agriculture, Forestry, and Bioresources, Research Institute of Agriculture and Life Sciences, College of Agriculture and Life Sciences, Seoul National University, Seoul 08826, Republic of Korea

**Keywords:** herbicides, nutritive value, safety, weed control, yield

## Abstract

The scarcity of effective selective herbicides has made weed management a limiting factor for alfalfa yield and large-scale cultivation. This study aimed to preliminarily screen selective herbicides with acceptable safety to alfalfa and establish an effective weed management program based on sequential pre- and post-emergence herbicide applications through greenhouse screening and multi-year field trials. The greenhouse herbicide safety evaluation showed that, among the 22 herbicides tested, the post-application of 2 pre-emergence herbicides (s-metolachlor and prodiamine) and 5 post-emergence herbicides (benazolin, bentazon, fluazifop-p, imazethapyr, and nicosulfuron) showed a relatively higher survival rate (~4× the standard dose) on two alfalfa cultivars through assessing visual efficacy, plant height, and dry biomass per plant. However, even herbicides that result in relatively high plant survival can produce induce significant growth inhibition and biomass reduction when applied at high doses. Those herbicides were further tested under field conditions (2020–2023) for evaluation of weed control efficiency and alfalfa plant biomass yield potential and nutritive values. Among the herbicide application regimes, the pre-application of prodiamine followed by post-application of bentazon or fluazifop-p showed the most effective weed control efficacy (70–85% reduction of weed biomass in the alfalfa plot), which was greater than the reductions achieved with all single-herbicide applications (24–48%) and sequential application of s-metolachlor followed by the five post-emergence herbicides (25–46%). Additionally, the optimized combinations had no significant effect on the 1st and subsequent alfalfa plant height compared to that of alfalfa in weed-free control. No significant reductions in alfalfa yield or nutritive value were observed in the herbicide-treated plots treated with prodiamine followed by bentazon or fluazifop-p compared with the weed-free control. Collectively, sequential application combining pre- (prodiamine) and post-emergence (bentazon or fluazifop-p) herbicides shows effective weed control in alfalfa under the tested conditions, which provides a useful weed management program to enhance alfalfa forage production and nutritive values.

## 1. Introduction

Alfalfa (*Medicago sativa* L.) is a perennial leguminous forage crop with high nutritional value and is known as the queen of forages [1,2]. In recent years, growing demand for dairy and meat products has driven the rapid expansion of alfalfa cultivation in China; nevertheless, domestic alfalfa supply remains insufficient to meet market needs [3]. Weeds are one of the major challenges in alfalfa forage production. Weeds impact the stand and forage nutritive value of alfalfa crops by competing directly for nutrients, light, or water [4,5]. Herbicide application serves as an effective approach to reduce weed competition, enhance alfalfa establishment, and improve subsequent forage yield and nutritional quality of alfalfa [6,7]. For example, weed control using herbicide application increased alfalfa forage yield up to 39% compared to nontreated plots [8]. In an established alfalfa field, winter annual grasses and broadleaf weeds that emerge in the fall and resume growth in early spring can cause significant reduction in forage protein content (~20%) during the 1st cutting; however, after weed control by herbicide, the protein content increased to >27% [7,9]. While effective weed control may occasionally lower total alfalfa forage yield due to herbicide phytotoxicity or weed biomass removal, severe weed infestation in alfalfa fields can substantially reduce alfalfa productivity and forage quality [6,10]. Therefore, maintaining effective weed control while minimizing crop injury remains a critical challenge for alfalfa production.

Herbicides, including 4-(2,4-dichlorophenoxy) butyric acid (2,4-DB amine), 2-methyl-4-chlorophenoxyacetic acid (MCPA), bromoxynil, and imazethapyr, have been reported to be used for weed control in alfalfa fields [7,11,12,13]. However, some herbicides caused greater alfalfa injury and growth reduction [7,14]. To the authors’ knowledge, currently there are only two herbicides (2,4-DB and hexazinone) registered for annual broadleaf weed control in alfalfa fields in China. With the rapid expansion of alfalfa cultivation in China, the scarcity of effective herbicides for weed control in alfalfa has become increasingly prominent.

More importantly, previous studies have primarily focused on evaluating the crop safety or weed control efficacy of individual pre-emergence or post-emergence herbicides in alfalfa, whereas integrated herbicide programs have received considerably less attention [7,11,12]. Pre-emergence herbicides are generally effective in suppressing the initial flush of weed emergence during crop establishment; however, their residual activity is often insufficient to provide season-long weed control, allowing later-emerging weed cohorts to escape treatment [15,16,17]. In contrast, post-emergence herbicides effectively control emerged weeds but cannot prevent early-season weed interference during the critical period of crop establishment, when weed competition has the greatest impact on crop growth and final yield [18,19]. In addition, post-emergence herbicides frequently exhibit a relatively narrow weed control spectrum and their efficacy is strongly influenced by weed growth stage and environmental conditions [16,20]. Consequently, sequential application of pre- and post-emergence herbicides has the potential to provide broader-spectrum and season-long weed suppression by combining the complementary advantages of both application timings, thereby improving weed control efficacy while reducing weed escapes, minimizing crop injury, and preserving forage productivity [17]. Such sequential herbicide programs have been widely adopted in major agronomic crops, including maize, soybean, cotton, and cereal production systems, where they improve weed control consistency and delay the evolution of herbicide-resistant weed populations [17,20,21]. Moreover, no comprehensive study has been conducted to establish such an integrated herbicide program for alfalfa production in China.

In view of this, the objectives of this study were to (i) preliminarily screen herbicides with acceptable selectivity toward alfalfa from a diverse group of herbicides with different modes of action using a greenhouse pot experiment; (ii) further evaluate their weed control efficacies and alfalfa safety, including alfalfa biomass yield potential and nutritive values under actual field conditions; and (iii) finally establish an effective weed control program combining application of pre- and post-emergence herbicides for alfalfa field production.

## 2. Materials and Methods

### 2.1. Alfalfa Materials and Herbicides Used

Two commercial alfalfa (*M. sativa*) cultivars, ‘Sandeli’ and ‘Huaiyin’, purchased from the Beijing Rytway Seed Co., Ltd. (Beijing, China), were used in this study. A germination test on the two cultivars showed greater than 98% seed germination before sowing. In the present study, 22 herbicides were tested, including 4 pre-emergence and 18 post-emergence herbicides with various modes of action. Table 1 provides detailed information on herbicide common names, formulations, modes of action, standard application doses, and manufacturers of the 22 herbicides.

### 2.2. Experimental Design and Procedure

This study consisted of two experimental phases. First, a greenhouse pot experiment was conducted to preliminarily screen selective herbicides (22 herbicides with different modes of action) with acceptable safety to alfalfa. Although some herbicides were classified as pre-emergence herbicides based on their registered use patterns, all herbicides were foliar-applied to established alfalfa plants in the greenhouse experiment. Therefore, this experiment was designed to evaluate herbicide selectivity toward alfalfa rather than true pre-emergence crop safety, emergence, or establishment. Subsequently, a three-year field experiment was conducted to evaluate the weed control efficacy, alfalfa biomass production, and nutritive values of selected herbicides under different application schemes, including sole pre- or post-emergence herbicide applications and sequential application of a pre-emergence herbicide followed by a post-emergence herbicide.

#### 2.2.1. Greenhouse Pot Experiment

A pot experiment evaluating the safety of pre- or post-emergence herbicides on alfalfa cultivars (‘Sandeli’ and ‘Huaiyin’) was conducted in a greenhouse of Prataculture Science (32°23′ N, 119°25′ E, 12 m a.s.l.) at Yangzhou University, Yangzhou, Jiangsu Province, China, from August to October 2020. On 6 August 2020, seeds of the two alfalfa cultivars were sown in 200-well plastic pots (2.4 × 2.4 × 3.6 cm) with commercial horticultural potting soil (Yiyuan Agriculture and Forestry Ltd., Suzhou, China) and grown in the aforementioned greenhouse (25/20 °C with a 16/8 h photoperiod supplemented by an overhead sodium lamp). When the seedlings of each alfalfa cultivar reached the 2–3-true-leaf stage, they were transplanted into larger plastic horticultural pots (pot size: 16 cm height and 14 cm diameter) containing the same aforementioned potting soil (4–5 seedlings in a pot) and raised in the greenhouse up to the 5–6-true-leaf stage before herbicide treatment. Prior to herbicide application, each pot was thinned to contain 3 healthy alfalfa plants. All 22 herbicides were foliar-applied to the two alfalfa cultivar plants at three doses (1×, 2×, and 4× the standard dose) using a backpack sprayer with flat-fan nozzles (8004, TeeJet spraying system, Wheaton, IL, USA) to deliver 1000 L ha^−1^ at 145 kPa (Table 1). Each herbicide dose treatment contained three pots (3 plants in each pot). The herbicide-untreated controls consisted of alfalfa plants sprayed only with distilled water (*n* = 3 pots with 3 plants per pot). After treatment, all treated plants were returned to the greenhouse (same growing conditions as above) and arranged in a randomized complete block design (RCBD) (*n* = 3). Root irrigation was initiated at 24 h after herbicide application to avoid herbicide wash-off and allow full herbicide absorption by alfalfa plants. The plant height and visual efficacy of all treated plants were determined every two weeks after treatment. Visual efficacy was rated on a scale of 0 (plants were completely killed)–10 (no herbicide damage and identical plant growth status to untreated control) (Supplementary Table S1). Briefly, ratings of 1–3 indicate severe injury (widespread necrosis, heavily stunted growth, limited new leaf emergence); 4–6 represent moderate injury (obvious chlorosis, partial leaf necrosis, and detectable growth reduction); 7–9 indicate (mild leaf chlorosis or slight leaf malformation) [22,23]. Prior to alfalfa flowering (42 days after treatment), plants of both cultivars were harvested for determination of the final plant height and dry biomass (oven-dried at 65 °C until a constant weight was obtained). This greenhouse experiment evaluated the herbicide tolerance of established young alfalfa plants and was not designed to test seed germination safety or seedling emergence risk following true pre-emergence soil application. The herbicides identified as potentially safe for alfalfa in the present greenhouse pot experiment were further evaluated under field conditions in the subsequent experiment.

#### 2.2.2. Field Experiment

##### Field Site, Experimental Design, and Field Management

A three-year field experiment (2020–2023) was conducted to further evaluate the safety of selected herbicides (from Section 2.2.1) on alfalfa biomass yield potential and forage nutritive values, as well as weed control efficacy in alfalfa field plots at the Yangzhou University Pratacultural Science Experimental Station (32°40′ N, 119°40′ E, 10 m a.s.l.), Jiangsu Province, China. The experimental site has a typical humid subtropical climate. Meteorological data from the previous 30-year period (1991–2020) showed that the average annual temperature was 16.2 °C and the annual accumulated precipitation was 1100 mm. The field soil was characterized as sandy loam with a pH in the range of 5.8–6.8 during the study period. There were no previous crops cultivated at the current experimental field site. The three-year study was conducted at three different locations within the current field site. Prior to ‘Sandeli’ alfalfa sowing, the weeds in the field were controlled by application of glyphosate at 675 g a.e. ha^−1^ (Roundup^®^, AS, Monsanto Company, Shanghai, China). The field was then chisel-plowed, followed by manual seedbed preparation and fertilization (N-P-K: 15–15–15 at the rate of 650 kg ha^−1^).

Among the two cultivars evaluated in the greenhouse experiment, ‘Sandeli’ showed relatively better tolerance and more consistent performance under the selected herbicide treatments. Therefore, it was chosen for the subsequent field experiment. The three years of alfalfa field sowing (fall season) occurred on 28 October 2020, 30 October 2021, and 13 October 2022 (Table 2). In each year, ‘Sandeli’ alfalfa seeds were manually sown in each 1.0 × 1.0 m^2^ field plot at a rate of 600 seeds m^−2^. Each plot consisted of 4 rows of alfalfa with a 15-cm row spacing. The sowing depth was 10 mm for all plots. Plots were separated by a 0.5 m wide bare-soil buffer zone to reduce herbicide drift between adjacent treatments. Herbicide treatments included the sole application of 2 pre-emergence (prodiamine and s-metolachlor) and 5 post-emergence (benazolin, bentazon, fluazifop-p, imazethapyr, and nicosulfuron) herbicides, and sequential application of those pre- and post-emergence herbicides (Table 3). In each year, right after ‘Sandeli’ alfalfa seeds were sown, the 2 pre-emergence herbicides at their standard doses were applied to the corresponding plots using the same backpack sprayer and spray volume as in the greenhouse experiment (Table 3). There were 36 plots treated with the 2 pre-emergence herbicides. Among those plots, 6 plots were for the sole application of the two pre-emergence herbicides (*n* = 3 plots for each herbicide); the remaining 30 plots were for the sequential application of pre-emergence and post-emergence herbicides (Table 3). After 3 months in the following spring (a lower rate of weed emergence in alfalfa plots during the winter period), the 5 post-emergence herbicides at their standard doses were solely applied to 15 plots (*n* = 3 plots for each herbicide). Meanwhile, they were also applied to the 30 plots that had been pretreated with the two pre-emergence herbicides in the fall of the previous year (Table 3). In each year, two types of control plots were included: weed-free plots (hand weeding, *n* = 3) and untreated plots (no herbicide treatment, *n* = 3). All herbicide treatments and control plots were arranged in a randomized complete block design (RCBD) with three replicates for each treatment. Therefore, a total of 57 plots were included in each experimental year. The alfalfa plants in each plot were cut two times in May and August in each experimental year (Table 2). In each year, no additional post-emergence herbicide application was conducted after the first alfalfa cutting. The experiment was conducted independently in three consecutive years.

During the study period, the soil temperature of the alfalfa field plot was recorded by a HOBO Pendant data logger (HOBO U23 Pro, Onset Computer Corporation, Bourne, MA, USA) that was buried at 5 mm depth with a recording frequency of 10 min. One data logger was placed in the central area of a randomly selected replicated plot within each block to represent soil temperature conditions across the trial area. No insecticides or fungicides were applied during the three years of the study. No supplementary irrigation was provided during the whole study period, and all alfalfa plots were grown under rain-fed conditions.

##### Weed Species, Abundance, and Control Efficiency

To provide the baseline information on weed species and abundance at the current experimental field site and climate, a weed survey from herbicide-untreated plots was conducted 30–50 days after herbicide application in each year. The weeds in those plots were identified to the species level, and the relative abundance of each weed species in each plot was determined by dividing the number of individual weeds of the species by the total number of weeds.

To evaluate and compare the weed control efficiency resulting from the herbicide treatments in each year, prior to the 1st time of alfalfa harvest, all weeds present in each alfalfa plot (including the control plots) were carefully harvested at the soil surface. Their dry weights were measured after oven-drying at 105 °C for 30 min and 65 °C until a constant weight was obtained. Weed control efficiency (WCE) was calculated according to the following equation [Equation (1)]:

(1) $$WCE\;\%=\left(\frac{W_{c}\;-\;W_{t}}{W_{C}}\right)\times 100$$

where $W_{c}$ represents the mean weed dry biomass in weed-free control (hand weeding), and $W_{t}$ represents the weed dry biomass in the herbicide-treated plots. Higher WCE values indicate greater weed suppression.

##### Alfalfa Plant Height, Biomass Yield Analysis, and Nutritive Values

Following each alfalfa aboveground cutting, ten alfalfa plants from each plot were randomly selected to determine the average plant height (cm), and the plot mean was considered one experimental unit for statistical analysis. Alfalfa dry biomass in each plot (g plot^−1^) was determined by oven-drying all alfalfa plants at 105 °C for 1 h and 65 °C for 12 h (a constant weight was achieved), and further converted to dry biomass yield in kg ha^−1^. To evaluate the herbicidal safety on alfalfa from each cutting, the yield loss (YL) for each treatment was determined using the following equation [Equation (2)] [4,24]:

(2) $$YL\;\%=\left(\frac{Y_{wf}\;-\;Y_{t}}{Y_{wf}}\right)\times 100$$

where *Y_wf_* represents alfalfa dry biomass obtained under weed-free conditions (hand-weeded plot), and *Y_t_* represents the alfalfa dry biomass after weed control by herbicide application (herbicide-treated plot), respectively.

Additionally, the nutritive values, including acid detergent fiber (ADF), neutral detergent fiber (NDF), and crude protein (CP) of alfalfa in each plot were determined accordingly. The ADF and NDF were determined based on a nylon bag technique proposed by Zhou (2000) [25]. The detailed experimental procedures and analytical methods were summarized in a previous study [1]. Relative feed value (RFV) was calculated based on [Equation (3)] [26]:

(3) $$RFV=\frac{120}{NDF}\times \frac{88.9-0.779\times ADF}{1.29}$$

Yield loss and relative feed value were calculated independently for each biological replicate. The mean value for each treatment was obtained by averaging the three replicate-derived indices, rather than computing indices from averaged biomass, ADF, and NDF data.

The CP content was calculated by multiplying the total N, which was determined by an automatic Kjeldahl apparatus (Kjeltec 8400 Analyzer Unit, FOSS Analytical AB, Hoganas, Sweden), by 6.25.

### 2.3. Meteorological Data

During the study period, the meteorological data, including monthly minimum, maximum, and mean air temperature, and accumulated precipitation were recorded and provided by the Yangzhou Meteorological Administration. The long-term mean monthly air temperature and accumulated precipitation (1991–2020) were obtained from the National Meteorological Information Centre of China (http://data.cma.cn/) (10 December 2023) and used for comparison with the current season climates.

### 2.4. Statistical Analysis

All raw data obtained from the three years of the study were initially tested for normality using the Shapiro–Wilk test and for homogeneity of variance using Levene’s test. As the data met the assumptions of normality and homogeneity of variance, the data were then subjected to analysis of variance (ANOVA). A linear mixed-effects model was adopted (treatments and interactions as fixed effects; block and plot nested within block as random effects). For the greenhouse pot experiment, ANOVA was conducted to determine the effects of the factors (herbicide dose, herbicide tested, and alfalfa variety) and interactions (alfalfa visual efficacy, plant height, and dry weight). For the field experiment, ANOVA was conducted to determine the effects of herbicide application scheme, cutting time, and experimental year, as well as their interactions, on weed biomass, weed control efficacy, alfalfa plant height, dry biomass yield, yield loss, and nutritive values. Percentage response variables (including abundance of individual weed, YL, CP, ADF, and NDF) were directly subjected to ANOVA using raw values. Residual diagnostics were performed to verify approximate normality and homogeneity of variance. Untransformed means are presented in all tables and figures. When significant interactions among herbicide application scheme, cutting time, and experimental year were detected, treatment responses were further examined within individual years and cutting times. To elucidate the overall trend of treatments on alfalfa growth and nutritive parameters at two consecutive cutting times, data combining across the three years was conducted, with means presented individually for the two cutting times. Treatment means were separated and compared using Tukey’s post hoc test at *p* < 0.05. All statistical analyses were performed using R 4.3.2 [27].

## 3. Results

### 3.1. Greenhouse Experiment to Screen Potentially Safe Herbicides to Alfalfa

**Figure 1:**
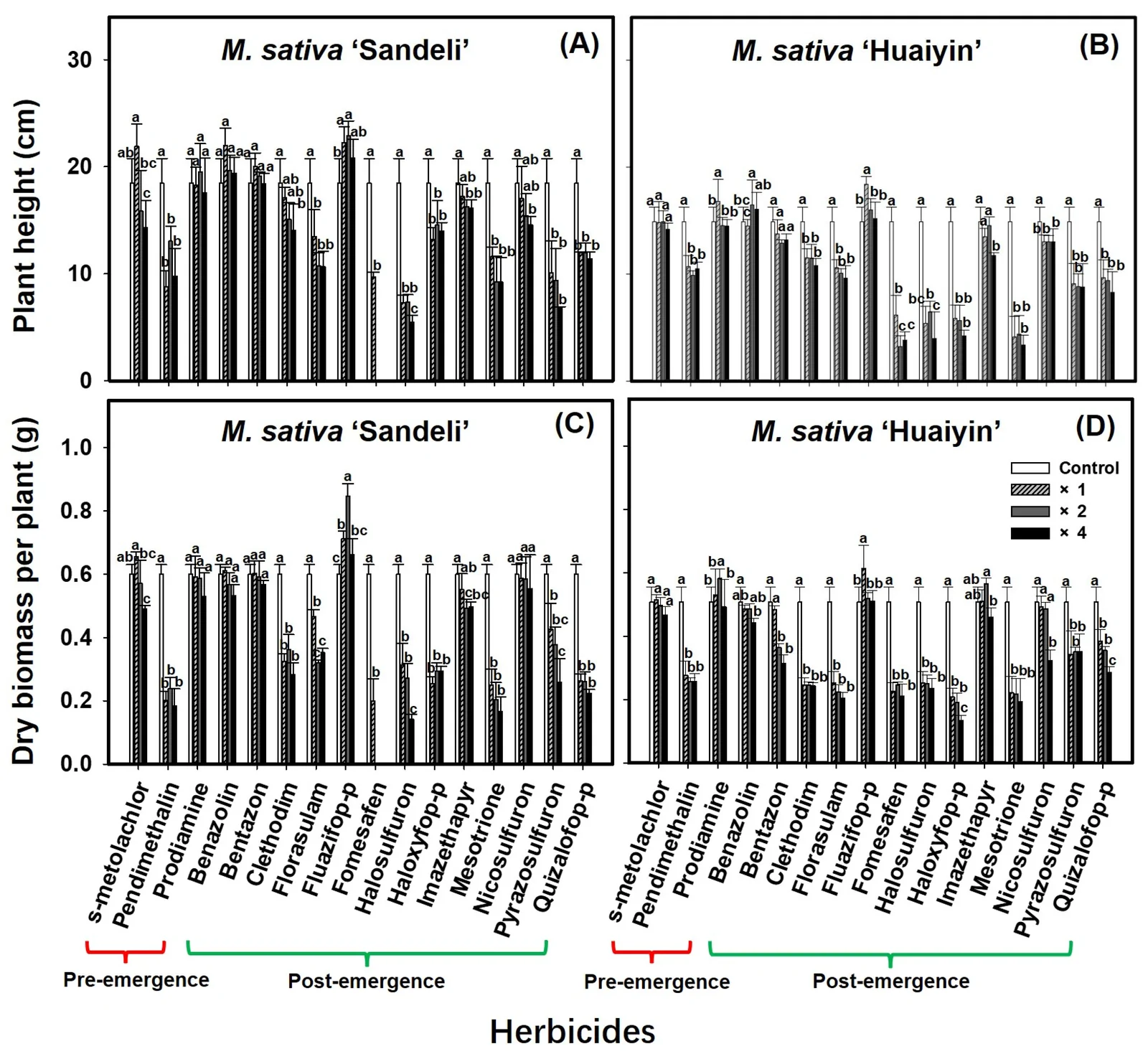
Greenhouse pot experiment for evaluation of the effect of post-application of 22 herbicides at 1×, 2×, and 4× the standard dose on plant height (cm) and dry biomass plant^−1^ of *Medicago sativa* ‘Sandeli’ (**A**,**C**) and *M. sativa* ‘Huaiyin’ (**B**,**D**). Four herbicides (e.g., clomazone, s-metolachlor) are registered as pre-emergence herbicides, and 18 herbicides (e.g., bentazon, benazolin) are registered as post-emergence herbicides (Table 1). In this figure, clomazone (pre-emergence herbicide) and the other 5 post-emergence herbicides (bispyribac, clopyralid, flumetsulam, oxyfluorfen, and quinclorac) are not included, as they completely killed the plants of the two alfalfa cultivars at any application dose. Means with the same letter are not significantly different according to Tukey’s post hoc tests at *p* < 0.05.

In the greenhouse pot study, the post-application of 22 herbicides (4 registered as pre-emergence and 18 as post-emergence herbicides) to the two alfalfa cultivars (‘Sandeli’ and ‘Huaiyin’) were evaluated for their safety. The herbicides tested, application dose, and alfalfa variety significantly affected the visual efficacy, plant height, and dry biomass of alfalfa (Table 4) compared with the untreated controls (Figure 1; Supplementary Table S1). Among the four pre-emergence herbicides tested, prodiamine (microtubule assembly inhibitor) showed the highest selectivity toward the two alfalfa varieties, maintaining plant growth (i.e., visual efficacy = 10) and biomass production ~4× the standard dose compared with the untreated controls (Figure 1; Supplementary Table S1). The s-metolachlor (cell division inhibitor) generally showed satisfactory safety on the two alfalfa varieties, although plant growth suppression was observed at the higher dose (>2×) on ‘Sandeli’ (Figure 1). Pendimethalin at all application doses resulted in significant visual damage (visual efficacy = 5–6) (Supplementary Table S1) and reduced plant height and biomass yield of both alfalfa varieties compared to the untreated controls (Figure 1). Regarding clomazone (IPP inhibitor), it completely killed the two alfalfa varieties at each application dose (Supplementary Table S1).

Among the 18 post-emergence herbicides tested, although benazolin (microtubule assembly inhibitor), bentazon (PS II inhibitor), and fluazifop-p (ACCase inhibitor) caused only slight visual injury (8–10) on the two alfalfa varieties (Supplementary Table S1), they did not affect the plant height and biomass yield of the alfalfa varieties up to 2× the standard dose (Figure 1). Two herbicides, imazethapyr and nicosulfuron, from the ALS inhibitor group were generally safe at their standard doses on the two alfalfa varieties (visual efficacy = 8–10). However, when the dose increased to 4× the standard dose, both the plant height and biomass yield of alfalfa varieties were significantly reduced (Figure 1). By contrast, other herbicides, even though applied at their standard doses, still caused significant plant growth suppression and biomass yield reduction. For example, haloxyfop-p, differing from fluazifop-p from the same ACCase inhibitor group, applied at any dose caused visual injury (5–7) on the two alfalfa varieties. Plant height and biomass yield of the two cultivars were reduced by >50% by this herbicide relative to the untreated controls (Figure 1). Florasulam and halosulfuron (ALS inhibitor) also significantly suppressed alfalfa growth and biomass yield. Additionally, bispyribac, clopyralid, flumetsulam, oxyfluorfen, and quinclorac were the least safe herbicides to alfalfa. Post-application of any of them at any application dose completely killed alfalfa plants within one week.

Based on the results presented above, seven herbicides (2 pre-emergence herbicides: s-metolachlor and prodiamine; 5 post-emergence herbicides: benazolin, bentazon, fluazifop-p, imazethapyr, and nicosulfuron) were further tested under field conditions for evaluation of weed control efficiency and alfalfa plant biomass yield potential and nutritive values.

### 3.2. Field Experiment to Evaluate Weed Control Efficiency and Alfalfa Safety with Selected Pre- and Post-Emergence Herbicides

#### 3.2.1. Meteorological Data During the Study

**Figure 2:**
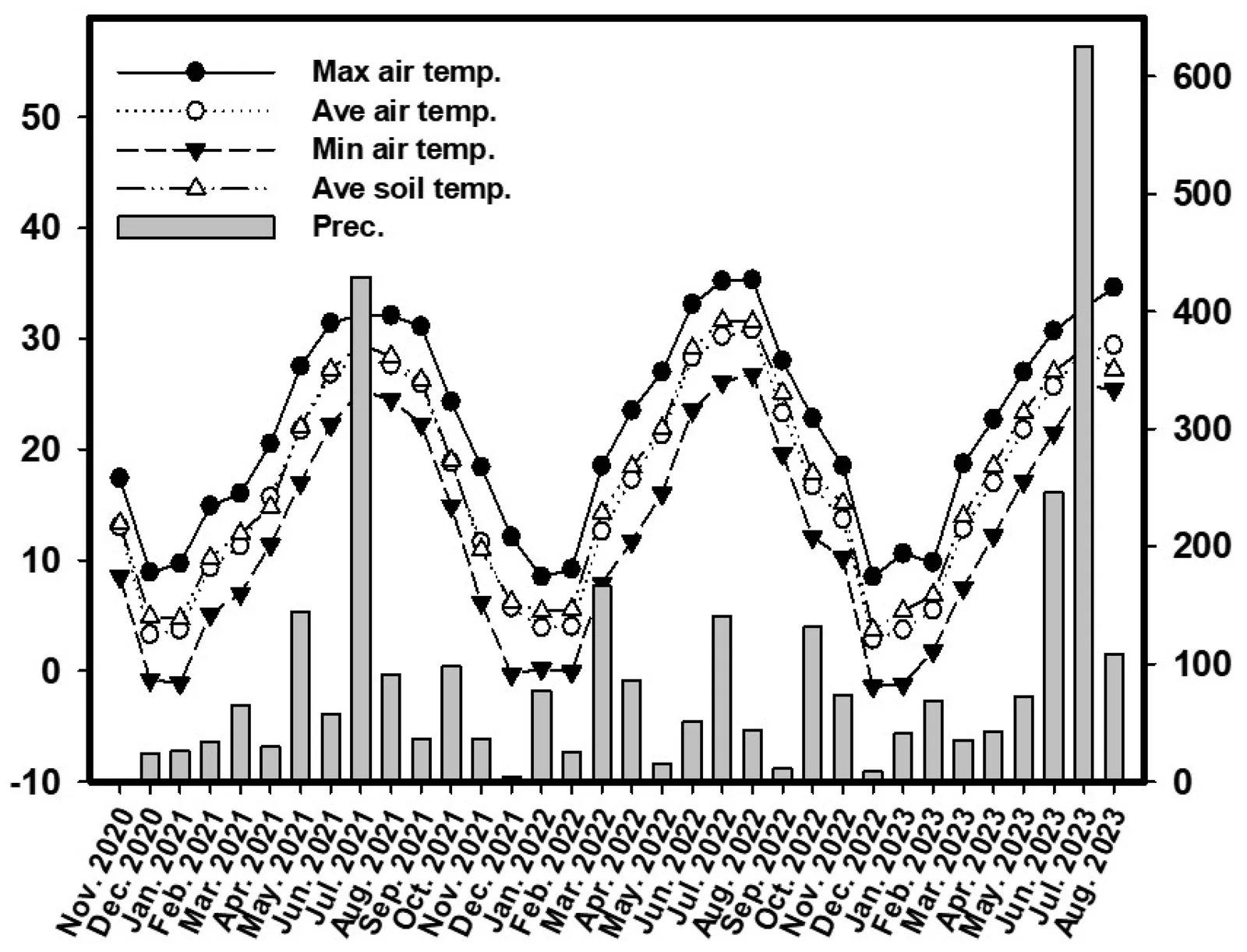
Monthly air temperature (max, min, and average), soil temperature, and accumulated precipitation during the three years of the study (Year 1: 2020–2021, Year 2: 2021–2022, and Year 3: 2022–2023).

In general, the mean air temperatures for the same months across the three years (2020–2023) were comparable (Figure 2), which was roughly consistent with the values for the long-term mean (1991–2020) (Supplementary Table S2). For example, the range of mean air temperatures in Year 1 was 3.3–28.3 °C, which was comparable to 3.9–30.8 °C in Year 2 and 2.8–28.8 °C in Year 3. Those temperature ranges were consistent with that of 2.9–28.4 °C in the previous 30-year period. The soil temperatures for the same months in the three years were also consistent (Figure 2). Regarding monthly precipitation, except for the greater precipitation of 429.2 mm in July in Year 1 (mean of the previous 30-year period: 210 mm), the overall precipitation for the other months in the three years was relatively consistent with the previous 30-year period (Supplementary Table S2). Taken together, the meteorological conditions during the three years of the study were relatively stable and consistent with the long-term meteorological data.

#### 3.2.2. Weed Control Efficiency

Significant treatment × year and treatment × cutting interactions were considered in statistical analysis, and treatment effects were interpreted within each experimental year and cutting period when interactions were significant. The weed survey from the alfalfa control plots across the three years of the study provided the baseline information on weed species and their relative abundances for the current study climate and region (Table 5). Overall, annual weed species were dominant at the current alfalfa field (abundance: about 97%). For example, the annual crabgrass [*Digitaria sanguinalis* (L.) Scop.] (mean across years: 31.8%), shepherd’s purse [*Capsella bursa-pastoris* (L.) Medik.] (mean across years: 24.6%) and common vetch (*Vicia sativa* L.) (mean across years: 17.0%) were the three most abundant weed species at the current field plots. One annual weed species, Japanese hop [*Humulus scandens* (Lour.) Merr.] (mean across years: 2.2%), and one perennial weed species, Chinese mugwort (*Artemisia argyi* Levl. et Vant.) (mean across years: 0.7%), were observed in the current field plots.

**Figure 3:**
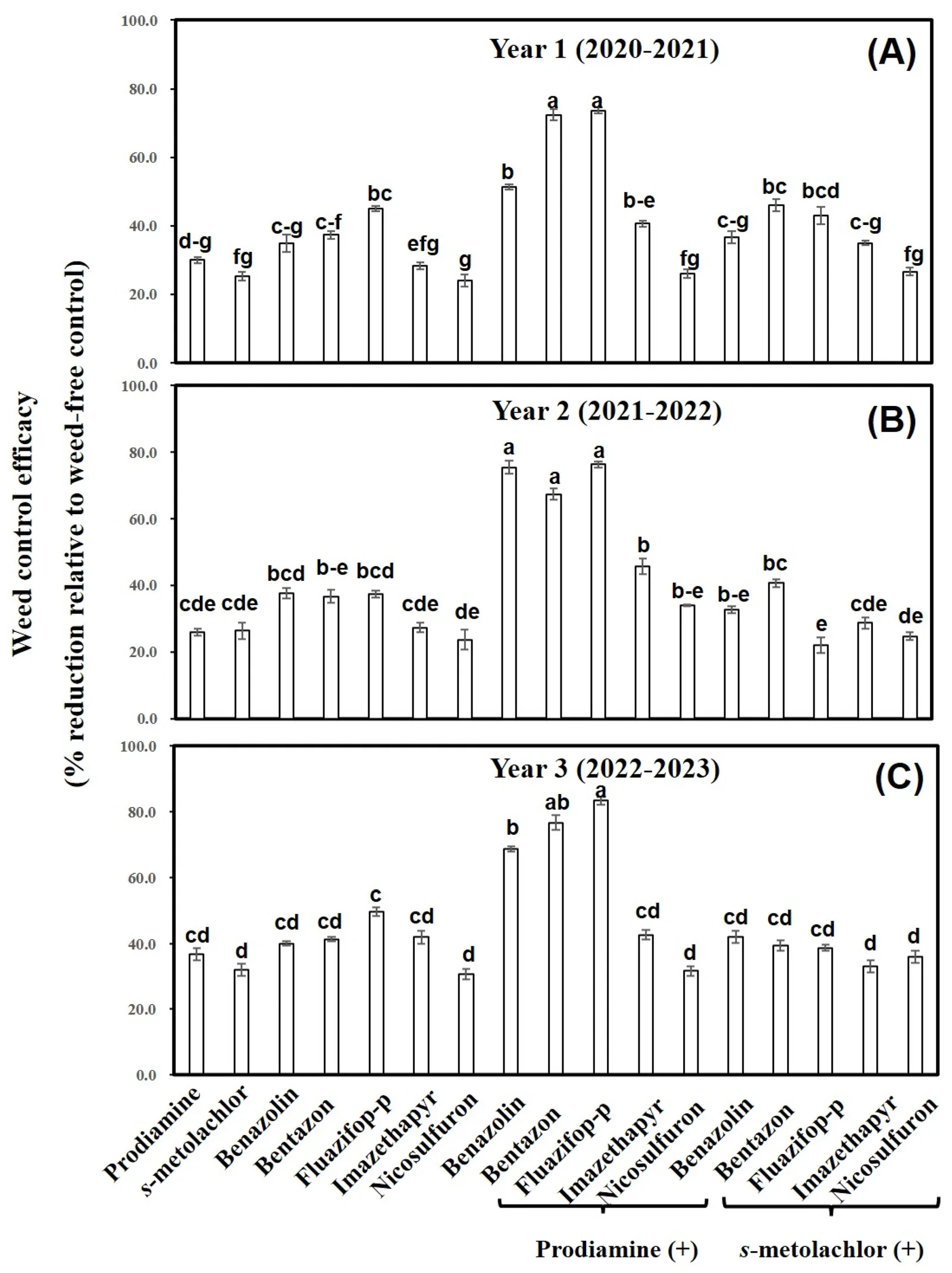
Weed control efficacy (% reduction of weed biomass compared to weed-free control: hand weeding) for *M. sativa* ‘Sandeli’ field plots treated with the screened herbicides at 1× the standard dose in Year 1 (2020–2021) (**A**), Year 2 (2021–2022) (**B**), and Year 3 (2022–2023) (**C**). Field herbicide treatments included the sole pre-application of 2 pre-emergence herbicides, prodiamine and s-metolachlor, post-application of 5 post-emergence herbicides, benazolin, bentazon, fluazifop-p, imazethapyr, and nicosulfuron, and sequential application combining the 2 pre-emergence herbicides and 5 post-emergence herbicides. Means with the same letter are not significantly different according to Tukey post hoc tests at *p* < 0.05.

Among the various herbicide treatments, sequential application of pre-emergence prodiamine + post-emergence bentazon and fluazifop-p herbicides showed the greatest weed biomass reduction among the tested herbicide programs (Figure 3). For example, sequential application of prodiamine + bentazon or fluazifop-p significantly reduced the percent weed dry biomass relative to the untreated controls (70–85%), which was greater than the values of 24–48% resulting from all the single herbicide applications, and 25–46% for the sequential application of pre-emergence s-metolachlor followed by the 5 post-emergence herbicides.

#### 3.2.3. Alfalfa Plant Safety and Nutritive Values

ANOVA showed that, except for plant height, all the other investigated parameters, including dry biomass, yield loss, crude protein, ADF, NDF, and RFV, were significantly affected by the three factors (Table 6). Significant interactions among herbicide application scheme, cutting time, and year were mainly observed for yield loss. However, plant height, dry biomass, and nutritive parameters showed similar response patterns across the three experimental years. To simplify and elucidate the overall treatment effects on alfalfa growth, yield, and nutritive values, data combining across the three years for the two different cutting times were conducted, with means reported accordingly (Table 7 and Table 8). Overall, among all herbicide treatments, pre-application of prodiamine followed by post-application of bentazon or fluazifop-p showed the greatest potential among the tested herbicide combinations in terms of alfalfa growth and forage nutritive values. As presented in Table 7 and Table 8, these two herbicide application combinations had no significant effect on the 1st (range of means: 45.56–52.37 cm) and subsequent alfalfa plant height (range of means: 48.27–48.46 cm) compared to the alfalfa plant height in weed-free controls (50.84 and 47.67 cm, respectively) at the two cutting times. Their dry weights derived from those two application combinations were similar to those of alfalfa dry weights in weed-free controls. No significant yield loss was observed in these herbicide-treated plots compared with weed-free controls. In contrast, the sequential application of pre-emergence herbicide s-metolachlor followed by the post-emergence herbicides significantly reduced alfalfa dry weights and caused yield losses ranging from 32.7–47.8% for the 1st cutting and 39.4–57.1% for the subsequent cutting, respectively (Table 7 and Table 8). With regard to alfalfa nutritive values, while variations in nutritive parameters among the treatments were observed, generally, CP, ADF, NDF, and RFV values in herbicide-treated plots were comparable with those in untreated controls, except for the reduced values observed following nicosulfuron application at the first cutting (Table 7).

## 4. Discussion

Weed management is a critical component of successful alfalfa production [12]. Not only do weeds emerging during the initial establishment stage affect alfalfa growth and development by competing for resources (e.g., light, water), but weeds infesting the late growing season can also have a notable negative impact on alfalfa yield and quality [28]. Therefore, integrated pre-emergence (targeting the soil seedbank) and post-emergence (controlling post-emerged weeds) herbicide weed management represents an effective strategy for crop production [29,30]. Here, it is worth noting that in broadleaf crops such as alfalfa, controlling emerged grass weeds with selective herbicides is relatively straightforward. However, managing broadleaf weeds in established alfalfa stands is far more challenging, which further complicates effective weed control. In this study, the two pre-emergence herbicides (s-metolachlor and prodiamine) and five post-emergence herbicides (benazolin, bentazon, fluazifop-p, imazethapyr, and nicosulfuron) with various modes of action were initially evaluated for alfalfa safety under greenhouse conditions. Based on this, a field trial was further conducted to evaluate the effects of various herbicide application regimes, applied alone or in combination, on alfalfa growth, yield, nutritive value, and weed control efficacy. Results from this study indicated that the pre-application of prodiamine (immediately after sowing) followed by post-application of bentazon or fluazifop-p (when weeds emerged in the following spring) provided effective control for grasses and broadleaf weeds, with no significant negative effects on alfalfa yield and nutritive value under the conditions evaluated. This approach may provide a promising weed management strategy to enhance alfalfa forage yield under similar agro-systems.

The application of pre-emergence herbicides is commonly used to manage the soil seedbank by forming a chemical barrier in the topsoil layer that kills germinating weeds prior to emergence, thereby reducing subsequent weed infestations [31,32,33]. In this study, the weed communities in the alfalfa fields were dominated by annual broadleaf species (approximately 70%). Application of pre-emergence herbicides (s-metolachlor and prodiamine) reduced the early-emerging weeds from the soil seedbank [34]; however, compared to s-metolachlor, prodiamine provided more effective control of annual and perennial broadleaf weeds, including shepherd’s purse, common vetch, and Japanese hop. This may be attributed to the much longer-lasting herbicide residual control efficacy of prodiamine (~5 months) than s-metolachlor (~3 months) [35,36]. Despite similar low phytotoxicity observed when these two pre-emergence herbicides were applied to alfalfa seedlings in the greenhouse, the two herbicides induced distinctly different impacts on alfalfa productivity under field pre-emergence soil application. S-metolachlor caused substantial alfalfa yield reduction in the field, especially when followed by post-emergence herbicides in sequence. This discrepancy is likely attributable to differences in herbicide uptake pathways and the developmental stage-dependent detoxification capacity of alfalfa. S-metolachlor is predominantly absorbed by germinating shoots and roots before emergence, making newly established seedlings particularly vulnerable owing to their limited capacity for glutathione-dependent herbicide metabolism [15,37,38]. Conversely, prodiamine primarily suppresses root meristem cell division through inhibition of microtubule polymerization but generally exhibits lower phytotoxicity to alfalfa seedlings under labeled use rates, resulting in relatively minor yield penalties [16]. Notably, the relatively high precipitation (165–268 mm) and low temperatures (2.9–9.8 °C) at the experimental site from December to March may have reduced the soil persistence of the two pre-emergence herbicides [36]. Nevertheless, subsequent application of the two post-emergence herbicides fluazifop-p or bentazon effectively controlled the late-emerging weeds in the late growing season, especially when they were applied sequentially with prodiamine. Additionally, this optimal application regime did not show any adverse effect on alfalfa plant height at the two consecutive cuttings. Overall, this sequential herbicide program exhibited good crop selectivity and maintained alfalfa growth, yield, and forage quality under the conditions of the present study. Besides effective weed control, lowered individual herbicide dosages can produce hormetic responses in crops: sublethal herbicide levels activate antioxidant metabolism, enhance nutrient uptake, and promote biomass accumulation [39,40]. Sequential application of these herbicides not only enhanced alfalfa yield but also had no adverse effect on its nutritive value. Although year-related interactions were detected for yield loss, the consistent responses of biomass production and forage quality among years indicate that the optimized herbicide programs provided stable effects under the tested field conditions. Interestingly, slightly negative yield loss values were observed in some optimized herbicide treatments, indicating marginally higher biomass than the weed-free control. These small differences should not be interpreted as a growth-promoting effect of herbicides, but may be attributed to experimental variation, including small plot-scale heterogeneity and possible disturbance associated with manual weed removal in weed-free plots.

Weed species present in fields across different regions can be highly diverse. The composition and distribution of weed species in the field, as well as their responses to herbicides, directly affect the efficacy of weed control [41,42]. The weeds present in this study are common in a smallholder farming system in China. In this farming system, weed communities in fields are relatively simple and low in species diversity. Weed control mainly focuses on common annual grass (e.g., crabgrass) and broadleaf weed (e.g., shepherd’s purse). By contrast, the large-scale agricultural systems in the U.S., Canada, and other regions are characterized by monocropping and the extensive planting of herbicide-resistant crops (e.g., glyphosate-resistant alfalfa). These agricultural practices have led to complex weed community structures and the evolution of herbicide-resistant superweeds [43,44]. For example, in the western U.S., control of a perennial plantain in alfalfa fields is extremely challenging due to its strong root system located deep within the soil [12,45]. However, in China, such problematic weeds are typically managed through manual removal. Additionally, although commercial adoption of glyphosate-resistant alfalfa has greatly facilitated weed management during alfalfa production, continuous use of glyphosate in glyphosate-resistant systems has resulted in the evolution of resistance in horseweed, kochia, and ryegrass [46]. These glyphosate-resistant weeds have further evolved into multiple herbicide-resistant superweeds under repeated selection pressure from herbicides with different modes of action, rendering them uncontrollable with existing herbicides [47,48,49]. In view of this, we would like to clarify that although sequential application of pre- and post-emergence herbicides achieved effective weed control under the present experimental conditions, the efficacy of this regime may be substantially reduced when applied to other weed species or herbicide-resistant weeds in fields located in certain regions [50,51]. 

Several limitations should be considered when interpreting the results of this study. The field experiments were conducted using only one alfalfa cultivar at a single location, and the relatively small plot size (1 × 1 m^2^) may limit the evaluation of biomass yield and weed control efficacy, as well as the extrapolation of these results to commercial-scale production systems. Weed communities and herbicide sensitivity may vary among regions, which could influence the effectiveness of these herbicide programs under different field conditions. Additionally, at the time of the post-emergence herbicide application in the field, the phenological stage of the alfalfa and the weeds was not recorded. Furthermore, this study mainly focused on the establishment year of alfalfa; therefore, additional multi-location and multi-year studies involving different cultivars and diverse weed communities are needed to further validate the long-term safety and effectiveness of these herbicide programs.

## 5. Conclusions

In conclusion, this study indicated that the sequential application of pre-emergence prodiamine followed by post-emergence bentazon or fluazifop-p achieves effective weed control in alfalfa under the tested experimental conditions. Specifically, the two post-emergence herbicides efficiently controlled the late-germinating annual grasses (e.g., crabgrass) and broadleaf weeds (e.g., shepherd’s purse, common vetch, Japanese hop) during the late growing season. This sequential herbicide treatment represents a promising experimental approach: under the tested conditions, it resulted in limited reductions in alfalfa total biomass, without adversely affecting forage nutritive traits including CP, ADF, NDF, and RFV. As noted above, the optimized herbicide regime exhibited excellent weed control efficacy in alfalfa under the current experimental conditions. However, its performance may differ across locations characterized by divergent weed community compositions, soil conditions, and climate. Further studies across different locations, cultivars, and production systems are needed to validate the long-term efficacy and safety of this herbicide program before broader recommendations can be made. We also would like to mention that the weed control efficacy in this study does not constitute a recommendation for commercial use in alfalfa fields and that use must comply with national registration and label requirements in China.

## Figures and Tables

**Table 1:** The detailed information on herbicide application times, mode of action, formulations, standard application doses, and manufacturers of the 22 herbicides used in this study.

Herbicides	Registered as Pre- or Post-Emergence Herbicide	Mode of Action ^a^	Formulation ^b^	g a.i. ha^−1^	Manufacturer
Clomazone	Pre	IPP	CS (360 g L^−1^)	648	Fumei Solid Plant Protectant Co., Ltd. (Shanghai, China)
s-metolachlor	Pre	CD	EC (960 g L^−1^)	1440	Xingyu Chemical Co., Ltd. (Hefei, China)
Pendimethalin	Pre	MA	EC (330 g L^−1^)	990	Longdeng Chemical Co., Ltd. (Rugao, China)
Prodiamine	Pre	MA	WG (65%)	780	Dongfang Agrochemical Co., Ltd. (Zhangjiagang, China)
Benazolin	Post	SA	SC (30%)	225	Futian Agrochemical Co., Ltd. (Nanjing, China)
Bentazon	Post	PS II	AS (480 g L^−1^)	1080	BASF Plant Protection Co., Ltd. (Nantong, China)
Bispyribac	Post	ALS	WP (20%)	45	Hulian Biopharmaceutical Co., Ltd. (Shanghai, China)
Clethodim	Post	ACCase	EC (120 g L^−1^)	63	Meilan Agricultural Development Co., Ltd. (Hefei, China)
Clopyralid	Post	SA	SG (75%)	101	Huimin Vanda Biological Technology Co., Ltd. (Jinan, China)
Florasulam	Post	ALS	SC (50 g L^−1^)	4	Dagong Agrochemical Co., Ltd. (Zhangye, China)
Fluazifop-p	Post	ACCase	EC (15%)	68	Wulong Pesticide Co., Ltd. (Wuhan, China)
Flumetsulam	Post	ALS	WG (80%)	60	Lawngreen Biotechnology Co., Ltd. (Zhengzhou, China)
Fomesafen	Post	PPO	AS (250 g L^−1^)	563	Best Agricultural Chemical Co., Ltd. (Suizhou, China)
Halosulfuron	Post	ALS	WG (75%)	34	Agricultural Technology Research Center Co., Ltd. (Nanjing, China)
Haloxyfop-p	Post	ACCase	EC (108 g L^−1^)	49	Flag Chemical Industry Co., Ltd. (Nanjing, China)
Imazethapyr	Post	ALS	AS (5%)	75	Cynda Chemical Co., Ltd. (Binzhou, China)
Mesotrione	Post	HPPD	OD (10%)	225	Shengbang Lvye Chemical Co., Ltd. (Jinan, China)
Nicosulfuron	Post	ALS	OD (40 g L^−1^)	42	Zhongbao Green Crop Technology Co., Ltd. (Langfang, China)
Oxyfluorfen	Post	PPO	EC (240 g L^−1^)	144	Nantong Jiahe Chemical Co., Ltd. (Nantong, China)
Pyrazosulfuron	Post	ALS	WP (10%)	23	Tianfeng Pesticide Chemical Co., Ltd. (Qiqihar, China)
Quinclorac	Post	SA	WP (50%)	225	Shengdan Biochemical Co., Ltd. (Hefei, China)
Quizalofop-p	Post	ACCase	EC (10%)	90	Best Agricultural Chemical Co., Ltd. (Suizhou, China)

^a^ IPP: isopentenyl pyrophosphate; CD: cell division; MA: microtubule assembly; PS II: photosystem II; ALS: acetolactate synthase; ACCase: acetyl-CoA carboxylase; SA: synthetic auxins; PPO: protoporphyrinogen oxidase; HPPD: 4-hydroxyphenyl pyruvate dioxygenase. ^b^ CS: capsule suspension; EC: emulsifiable concentrate; WG: water-dispersible granules; SC: suspension concentrate; AS: aqueous solution; WP: wettable powder; SG: soluble granules; OD: oil dispersion.

**Table 2:** Summary of the sowing and 1st and 2nd *M. sativa* (‘Sandeli’) cutting dates during the three years of the study (2020–2021, 2021–2022, and 2022–2023).

Year of Study	Sowing Date	1st Cutting Date	2nd Cutting Date
2020–2021	28 Oct. 2020	30 May 2021	24 August 2021
2021–2022	30 Oct. 2021	27 May 2022	9 August 2022
2022–2023	13 Oct. 2022	24 May 2023	19 August 2023

**Table 3:** Summary of the treatments for the sole application of pre- or post-emergence herbicides, combining pre- and post-emergence herbicides applications, and controls (weed-free and untreated) in this study.

Herbicide Types	Treatment	Application Scheme	Application Timing (Standard Dose)	No. of Treated Plots
Control groups	Weed-free (hand weeding)	-	-	3
	No herbicide-treated	-	-	3
Pre-emergence herbicide	Prodiamine	Sole application	Applied immediately after seed sown in fall	3
	s-metolachlor			3
Post-emergence herbicide	Benazolin		Applied when weeds emerged in the following spring	3
	Bentazon			3
	Fluazifop-p			3
	Imazethapyr			3
	Nicosulfuron			3
Pre- + Post-emergence herbicides	Prodiamine + Benazolin	Combining pre- and post-emergence herbicides applications	Pre-emergence herbicide applied immediately after seeds sown in fall; post-emergence herbicide applied when weeds emerged in the following spring	3
	Prodiamine + Bentazon			3
	Prodiamine + Fluazifop-p			3
	Prodiamine + Imazethapyr			3
	Prodiamine + Nicosulfuron			3
	s-metolachlor + Benazolin			3
	s-metolachlor + Bentazon			3
	s-metolachlor + Fluazifop-p			3
	s-metolachlor + Imazethapyr			3
	s-metolachlor + Nicosulfuron			3

**Table 4:** The ANOVA for factor effects (herbicide dose, herbicide tested, and alfalfa variety) and interactions on visual efficacy (VE), plant height (PH), and dry biomass (DB) of the two *M. sativa* varieties (‘Sandeli’ and ‘Huaiyin’) in the greenhouse pot experiment.

Source of Variation		VE	PH	DB
	Df	*p*	*p*	*p*
Dose (D)	2	***	***	***
Herbicide tested (H)	21	***	***	***
Variety (V)	1	***	***	***
D × H	42	***	***	***
D × V	2	***	NS	NS
H × V	21	***	***	***
D × H × V	42	***	*	***

NS: Not significant; * and *** represent significant at 0.05 and 0.001 probability level, respectively.

**Table 5:** Weed species present in *M. sativa* (‘Sandeli’) control plots (*n* = 3, no herbicide-treated plots) and their relative abundances across the three years of the study (2020–2021, 2021–2022, and 2022–2023).

Weed Species	Family	Life Form	Abundance (%) of Individual Weed			Mean (%)
			2020–2021	2021–2022	2022–2023	
Crabgrass [*Digitaria sanguinalis* (L.) Scop.]	Poaceae	Annual	30.6 (36.0) ^a^	35.1 (52.7)	29.7 (24.4)	31.8
Shepherd’s purse [*Capsella bursa-pastoris* (L.) Medik.]	Brassicaceae	Annual	28.5 (33.6)	14.1 (21.2)	31.1 (25.5)	24.6
Common vetch (*Vicia sativa* L.)	Fabaceae	Annual	12.2 (14.4)	19.9 (29.9)	18.9 (15.5)	17.0
Carolina geranium [*Geranium carolinianum* L.]	Geraniaceae	Annual	9.2 (10.8)	14.2 (21.3)	6.4 (5.2)	9.9
Sticky chickweed [*Cerastium glomeratum* Thuill.]	Caryophyllaceae	Annual	7.2 (8.4)	5.4 (8.1)	7.2 (5.9)	6.6
Annual fleabane [*Erigeron annuus* (L.) Pers.]	Asteraceae	Annual	2.0 (2.4)	5.9 (8.9)	4.3 (3.5)	4.1
Sun spurge (*Euphorbia helioscopia* L.)	Euphorbiaceae	Annual	4.1 (4.8)	2.9 (4.4)	2.4 (2.0)	3.1
Japanese hop [*Humulus scandens* (Lour.) Merr.]	Cannabaceae	Annual	4.1 (4.8)	2.5 (3.8)	-	2.2
Chinese mugwort (*Artemisia argyi* Levl. et Vant.)	Asteraceae	Perennial	2.0 (2.4)	-	-	0.7

^a^ The data in parentheses represent the mean number of individual weed species present in the control plot.

**Table 6:** The ANOVA for factor effects (cutting times, herbicide application scheme, and experimental year) and interactions on plant height (PH), dry biomass (DB), yield loss (YL), crude protein (CP), acid detergent fiber (ADF), neutral detergent fiber (NDF), and relative feed value (RFV) of herbicide treatment in alfalfa (‘Sandeli’) under field conditions.

Source of Variation		PH	DB	YL	CP	ADF	NDF	RFV
	Df	*p*	*p*	*p*	*p*	*p*	*p*	*p*
Cutting time (CT)	1	NS	***	*	***	***	***	***
Herbicide application scheme (HAS)	18	***	***	***	*	**	**	**
Year (Y)	2	NS	**	***	***	**	**	**
CT × HAS	18	NS	NS	***	NS	**	**	**
CT × Y	2	NS	NS	**	NS	***	***	***
HAS × Y	36	NS	NS	*	NS	NS	NS	NS
CT × HAS × Y	36	NS	NS	***	NS	NS	NS	NS

NS: Not significant; *, **, and *** represent significant at 0.05, 0.01, and 0.001 probability level, respectively.

**Table 7:** Mean plant height (cm), dry biomass (DB) (kg ha^−1^), yield loss (YL) (%), crude protein (CP) (%), acid detergent fiber (ADF) (%), neutral detergent fiber (NDF) (%), and relative feed value (RFV) of the 1st alfalfa cutting for the different treatments under field conditions across the three years of the study (2020–2021, 2021–2022, and 2022–2023). Different letters within each column indicate significant different values determined by Tukey post hoc tests at *p* < 0.05.

Treatment	PH	DB	YL	CP	ADF	NDF	RFV
Control 1	50.84 a	4945.25 a	-	23.67 a	18.32 c–f	35.57 ab	228.12 ab
Control 2	38.63 f	3488.28 cd	29.46 d	22.72 ab	19.32 b–d	34.28 a–d	201.95 c–e
Pre 1	43.50 c–f	3540.33 cd	28.41 de	23.68 a	19.07 b–d	33.26 b–g	226.63 ab
Pre 2	40.96 d–f	3182.13 cde	35.65 cd	22.51 ab	18.12 c–f	35.70 ab	229.77 ab
Post 1	47.22 a–c	4540.20 ab	8.19 f	22.79 a	18.72 c–e	35.05 ab	224.42 ab
Post 2	44.35 b–e	4686.33 a	5.24 fg	23.05 a	19.57 b–d	34.09 a–d	225.25 ab
Post 3	52.04 a	4746.00 a	4.03 fg	22.91 a	20.59 ab	34.85 a–c	220.65 a–c
Post 4	40.20 d–f	3822.87 bc	22.70 e	22.98 a	20.07 bc	35.85 a	221.5 a–c
Post 5	30.78 g	2952.65 de	40.29 bc	19.51 c	16.87 ef	31.79 e–h	192.95 e
Pre 1 + Post 1	49.21 ab	4681.25 a	5.34 fg	22.78 ab	17.95 d–f	33.75 a–e	211.32 b–e
Pre 1 + Post 2	45.56 a–c	4929.33 a	0.32 g	23.47 a	19.34 b–d	32.36 c–h	220.21 a–c
Pre 1 + Post 3	52.37 a	5032.95 a	−0.05 g	22.97 a	19.97 bc	33.39 a–g	222.72 ab
Pre 1 + Post 4	44.13 b–e	3345.80 cde	32.34 d	23.75 a	19.22 b–d	33.64 a–f	209.97 b–e
Pre 1 + Post 5	38.96 ef	3365.15 cde	31.95 d	20.38 bc	20.18 a–c	34.54 a–d	207.44 b–e
Pre 2 + Post 1	39.98 d–f	2743.37 e	44.53 ab	23.85 a	19.27 b–d	30.07 h	239.46 a
Pre 2 + Post 2	39.60 ef	3197.20 cde	35.35 cd	23.46 a	22.51 a	30.99 f–h	229.17 ab
Pre 2 + Post 3	42.74 c–f	3329.87 cde	32.67 d	23.69 a	18.06 c–f	31.32 d–h	223.89 ab
Pre 2 + Post 4	42.53 c–f	2975.17 de	39.84 bc	23.54 a	18.01 c–f	30.87 gh	200.59 de
Pre 2 + Post 5	26.37 g	2581.30 e	47.80 a	19.36 c	16.20 f	31.37 d–h	198.85 e

Control 1: weed-free (hand weeding); Control 2: no herbicide treatment; Pre 1: prodiamine; Pre 2: s-metolachlor; Post 1 to 5: benazolin, bentazon, fluazifop-p, imazethapyr, and nicosulfuron, respectively.

**Table 8:** Mean plant height (cm), dry biomass (DB) (kg ha^−1^), yield loss (YL) (%), crude protein (CP) (%), acid detergent fiber (ADF) (%), neutral detergent fiber (NDF) (%), and relative feed value (RFV) of the 2nd alfalfa cutting for the different treatments under field conditions across the three years of the study (2020–2021, 2021–2022, and 2022–2023). Different letters within each column indicate significant different values determined by Tukey post hoc tests at *p* < 0.05.

Herbicides	Height	DW	Yield Loss	CP	ADF	NDF	RFV
Control 1	47.67 ab	3386.40 a	-	20.63 a	18.28 b–d	34.28 b	203.06 a
Control 2	42.39 b–g	1663.75 gh	50.87 a	19.46 a	18.75 a–c	37.79 ab	166.77 b
Pre 1	43.49 a–f	2705.80 cde	20.10 cde	20.42 a	18.31 c–d	36.55 ab	193.92 ab
Pre 2	43.71 a–f	2348.15 ef	30.66 bc	20.21 a	19.07 a–c	35.58 ab	197.76 ab
Post 1	40.58 d–g	2849.00 bcd	15.87 def	18.82 a	19.11 a–c	40.92 a	188.31 ab
Post 2	41.33 c–g	2543.25 de	24.90 cd	19.71 a	20.93 a	40.67 a	176.93 ab
Post 3	46.71 a–c	3073.75 abc	9.23 efg	20.89 a	18.70 a–c	36.72 ab	194.96 ab
Post 4	42.12 c–g	2886.50 bcd	14.76 def	20.24 a	18.75 a–c	37.41 ab	186.96 ab
Post 5	38.78 fg	2372.10 ef	29.95 bc	19.73 a	15.76 d	38.65 ab	180.16 ab
Pre 1 + Post 1	48.26 a	2885.50 bcd	14.79 def	19.12 a	17.10 cd	40.04 ab	175.93 ab
Pre 1 + Post 2	48.27 a	3380.65 a	0.16 g	20.27 a	18.95 a–c	36.14 ab	194.14 ab
Pre 1 + Post 3	48.46 a	3399.25 a	−1.09 g	20.56 a	19.13 ab	35.85 ab	194.25 ab
Pre 1 + Post 4	44.43 a–e	3198.55 ab	5.55 fg	20.35 a	19.45 a–c	36.00 ab	190.91 ab
Pre 1 + Post 5	41.97 c–g	2992.70 abc	11.63 ef	20.31 a	20.24 ab	38.78 ab	179.75 ab
Pre 2 + Post 1	40.28 d–g	1574.50 h	53.51 a	18.47 a	19.45 a–c	38.45 ab	178.88 ab
Pre 2 + Post 2	37.04 g	1452.65 h	57.10 a	17.98 a	18.58 a–c	39.51 ab	175.77 ab
Pre 2 + Post 3	45.12 a–d	2051.00 fg	39.43 b	20.41 a	17.52 cd	36.45 ab	192.54 ab
Pre 2 + Post 4	39.24 e–g	1655.00 gh	51.13 a	19.89 a	20.40 a–c	40.05 ab	174.94 ab
Pre 2 + Post 5	42.37 b–g	1476.00 h	56.41 a	18.46 a	18.98 a–c	40.21 ab	172.77 ab

Control 1: weed-free (hand weeding); Control 2: no herbicide treatment; Pre 1: prodiamine; Pre 2: s-metolachlor; Post 1 to 5: benazolin, bentazon, fluazifop-p, imazethapyr, and nicosulfuron, respectively.

## Data Availability

Data will be made available by the authors on request.

## Supplementary Materials

The following supporting information can be downloaded at: https://www.mdpi.com/article/10.3390/plants15162461/s1, Table S1: Visual efficacy of the 22 herbicides tested on *M. sativa* cultivars (‘Sandeli’ and ‘Huaiyin’) at 21 days after herbicide treatments. Table S2: Monthly average air temperature (°C) and accumulated precipitation (mm) for the past 30-year mean (1991–2020) at Yangzhou, China.
