# Supplementary material for: Combined Pre- and Post-Emergence Herbicides for Effective Weed Control in Alfalfa (Medicago sativa L.)
Source: Plants (Basel). 2026 Aug 13;15(16):2461. doi: 10.3390/plants15162461 (PMC13516947; doi:10.3390/plants15162461)
Supplement: Supplementary file 1 [file plants-15-02461-s001.zip › plants-4443628-supplementary.pdf]

Article

Combined Pre- and Post-Emergence Herbicides for Effective Weed Control in Alfalfa (*Medicago sativa* L.)

Xin-Ran Bao [1], Yang Gao [1], Jin-Won Kim [2], Min-Jung Yook [3], Do-Soon Kim [3] and Chuan-Jie Zhang [1,*]

[1] College of Animal Science and Technology, Yangzhou University, Yangzhou 225009, China; 19906151359@163.com (X.-R.B.); xx_gaoyang@163.com (Y.G.)
[2] National Institute of Agricultural Sciences, Rural Development Administration, Wanju 55365, Jeollabukdo, Republic of Korea
[3] Department of Agriculture, Forestry, and Bioresources, Research Institute of Agriculture and Life Sciences, College of Agriculture and Life Sciences, Seoul National University, Seoul 08826, Republic of Korea
* Correspondence: chuanjiezhang@yzu.edu.cn (C.-J. Z.)

Supplementary Materials

Table S1. Visual efficacy of the 22 herbicides tested on *M. sativa* cultivars ('sandeli' and 'huaiyin') at 21 days after herbicide treatments.

**Herbicide tested**	***M. sativa* 'Sandeli'**			***M. sativa* 'Huaiyin'**		
	**×1**	**×2**	**×4**	**×1**	**×2**	**×4**
Clomazone	0	0	0	0	0	0
s-metolachlor	10	8	8	10	10	8
Pendimethalin	6	5	5	6	6	5
Prodiamine	10	10	10	10	10	10
Benazolin-*ethyl*	10	9	8	10	8	8
Bentazon	9	9	8	9	8	8
Bispyribac-sodium	0	0	0	0	0	0
Clethodim	7	6	6	8	7	5
Clopyralid	0	0	0	0	0	0
Florasulam	6	5	5	4	3	4
Fluazifop-*p*	10	10	9	10	10	9
Flumetsulam	0	0	0	0	0	0
Fomesafen	5	0	0	0	0	0
Halosulfuron-*methyl*	3	3	3	4	3	3
Haloxyfop-*p*	6	6	5	7	6	5
Imazethapyr-ammonium	9	9	8	9	9	8
Mesotrione	5	4	3	6	5	4
Nicosulfuron	10	9	8	10	9	8
Oxyfluorfen	0	0	0	0	0	0
Pyrazosulfuron-*ethyl*	6	6	6	5	5	5
Quinclorac	0	0	0	0	0	0
Quizalofop-*p*	7	5	5	8	8	6

Table S2. Monthly average air temperature (°C) and accumulated precipitation (mm) for the past 30-year mean (1991–2020) at Yangzhou, China[a].

Locations	30-year average [a]											
	Jan.	Feb.	Mar.	Apr.	May	Jun.	Jul.	Aug.	Sep.	Oct.	Nov.	Dec.
Yangzhou	Mean monthly air temperature (°C)											
	2.9	5.2	9.8	15.9	21.4	24.9	28.4	27.9	23.5	17.8	11.5	5.1
	Accumulated monthly precipitation (mm)											
	50.5	49.1	75.8	69.6	86.2	165.2	210.3	174.2	74.3	54	54.1	36.6

[a] The meteorological date were obtained from National Meteorological Information Centre of China (http://data.cma.cn/).
